# Constructing a Prognostic Model of Uterine Corpus Endometrial Carcinoma and Predicting Drug-Sensitivity Responses Using Programmed Cell Death-Related Pathways

**DOI:** 10.7150/jca.92201

**Published:** 2024-03-31

**Authors:** Jingwen Meng, Chen Zong, Meixia Wang, Yu Chen, Shaojie Zhao

**Affiliations:** 1 Wuxi Maternity and Child Health Care Hospital, Affiliated Women's Hospital of Jiangnan University, Wuxi, China.; 2 Department of Gynecology and Obstetrics, Wenzhou Hospital of Integrated Traditional Chinese and Western Medicine, Wenzhou, China.

**Keywords:** Programmed cell death, Uterine Corpus Endometrial Carcinoma, prognostic signature

## Abstract

**Background:** Uterine Corpus Endometrial Carcinoma (UCEC) is the most common type of cancer that develops in the uterus, specifically originating from the endometrium, the inner lining of the uterus. Programmed cell death (PCD) is a highly regulated process that eliminates damaged, aged, or unwanted cells in the body. Dysregulation of PCD pathways can contribute to the formation and progression of various cancers, including UCEC.

**Methods:** Fourteen PCD pathways (autophagy-dependent cell death, alkaliptosis, apoptosis, cuproptosis, entotic cell death, ferroptosis, immunogenic cell death, lysosome-dependent cell death, MPT-driven necrosis, necroptosis, netotic cell death, oxeiptosis, parthanatos, and pyroptosis) were involved in building a prognostic signature. The model was trained and tested using data from the TCGA-UCEC and validated with the GSE119041 dataset.

**Results:** A 12-gene PCD signature (DRAM1, ELAPOR1, MAPT, TRIM58, UCHL1, CDKN2A, CYFIP2, AKT2, LINC00618, TTPA, TRIM46, and NOS2) was established and validated in an independent dataset. UCEC patients with a high PCD score (PCDS) exhibited worse prognosis. Furthermore, PCDS was found to be associated with immune related cells and key tumor microenvironment components through multiple methods. It was observed that UCEC patients with a high PCD score may not benefit from immunotherapy, but some chemo drugs like Bortezomib may be useful.

**Conclusion:** In conclusion, a novel PCD model was established by comprehensively analyzing diverse cell death patterns. This model accurately predicts the clinical prognosis and drug sensitivity of UCEC. The findings suggest that the PCD signature can serve as a valuable tool in assessing prognosis and guiding treatment decisions for UCEC patients.

## Introduction

Uterine Corpus Endometrial Carcinoma (UCEC, also known as Endometrial adenocarcinoma) is the most common malignant tumor of the female reproductive tract in the United States. An estimated 65,950 new cases and 12,550 deaths of uterine cancer are expected to occur in 2022. Treatment of endometrial cancer involves multiple approaches, including surgery, chemotherapy, radiation therapy and targeted therapies, depending on the histopathologic evaluation and clinical presentation 1.

Programmed cell death a rigorously regulated process that eliminates damaged, aged, or unwanted cells in the body. It plays a critical role in maintaining tissue homeostasis and preventing cancer development 2. Dysregulation of programmed cell death pathways is implicated in the formation and progression of various cancers, including UCEC 3.

There are several key pathways involved in programmed cell death, including autophagy-dependent cell death (ADCD), alkaliptosis, apoptosis, cuproptosis, entotic cell death (entosis), ferroptosis, immunogenic cell death (ICD), lysosome-dependent cell death (LDCD), MPT-driven necrosis (MPTDN), necroptosis, netotic cell death (NETosis), oxeiptosis, parthanatos and pyroptosis 4-7. Among these, cuproptosis, a copper-dependent regulated non-apoptotic form of cell death, triggers oxidative stress and disrupts the ubiquitin-proteasome system to induce cell death 7, 8.

Understanding the dysregulation of these programmed cell death-related pathways in UCEC can provide insights into the underlying mechanisms of tumor development and progression. It can also potentially lead to the development of therapeutic strategies targeting these pathways for the treatment of UCEC.

## Materials and Methods

### Gene collection and datasets

Based on relevant studies, we included 14 programmed death-related pathways. Genes were collected from the Molecular Signatures Database (MSigDB), The Human Gene Database (GeneCards), Kyoto encyclopedia of genes and genomes (KEGG), or through manual review. In brief, there are three main steps (see **Supplementary Table S1** for details). The first step involved utilizing authoritative databases, such as the Human Autophagy Database (HADb, http://www.autophagy.lu/) for ADCD, FerrDb V2 (http://www.zhounan.org/ferrdb/current/) 9 for ferroptosis. In the second step, in the absence of a relevant database, we used MSigDB and KEGG to construct the gene set, for example, apoptosis gene set consisted of ALCALA_APOPTOSIS, GOBP_APOPTOTIC_SIGNALING_PATHWAY, HALLMARK_APOPTOSIS, REACTOME_APOPTOSIS and WP_APOPTOSIS (the gene set names in MSigDB). In the third step, since some pathways are relatively new, we manually collected genes from related articles, such as cuproptosis 7, 8.

For public data, we collected information on 539 UCEC patients and 35 controls from TCGA-UCEC (downloaded from GDC Data Portal: https://portal.gdc.cancer.gov/), and 50 UCEC cases in GSE119041 10, which was generated on the GPL15048 platform in the Gene Expression Omnibus (GEO) database (**Table 1**). An oncoplot was created to present descending order of mutations with the R package “maftools” (version 2.12.0). In TCGA, gene expression was preprocessed by STAR method and then transformed into transcripts per million (TPM). The further preprocessing steps for the RNA-seq data obtained from TCGA-UCEC involved the removal of samples lacking follow-up information, conversion of ENSEMBL IDs to gene symbols, and elimination of genes with a proportion exceeding 50% across all samples. Regarding the GEO cohort, the following processing steps were applied: removal of samples lacking clinical follow-up information, elimination of probes corresponding to multiple genes, and utilization of the mean expression value for multiple genes.

### Differential gene analysis and enrichment analysis

Raw transcriptome counts data of 539 UCEC patients and 35 controls in the TCGA-UCEC cohort were analyzed. The R Package edgeR* (*version 3.42.4) was utilized to screen out differentially expressed genes (DEGs) with the criterion of the false discovery rate (FDR) < 0.05 and an absolute value of log2 fold change (log2 FC) > 1. R package clusterProfiler 11 was used to identify possible biological pathways based on DEGs.

### Programmed cell death score modeling and validation

Univariate Cox regression was utilized to assess the impact of specific genes on the survival status of UCEC. To avoid omissions, we adjusted the cut-off P-value to 0.1. The least absolute shrinkage and selection operator (LASSO) Cox regression method was further used to refine the candidate genes to construct the optimal signature. we selected the “lambda. min” value with R package “glmnet”. Ultimately, the model exported the cell death index (PCDS) for each patient by the formula below: PCDS =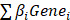 . 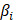
denotes the risk coefficient and 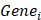 refers the expression of each gene. To make plots more intuitionistic, we applied a linear transformation to PCDSs. This involved subtracting the minimum PCDS value from each score and dividing by the maximum, scaling the scores to a 0-1 range. According to the median value of the PCDS, we divided UCEC patients into low-PCDS and high-PCDS groups. We used the “stats” package to perform principal component analysis (PCA) and used the “survival” and “survminer” package for Kaplan-Meier analysis to investigate the relationship between OS time and PCDS.

### Establishment and application of programmed cell death prognostic signature

Clinical features (age, T, N, and M stage) were combined with PCDS to establish a prognostic nomogram via multivariable Cox and stepwise regression analyses. The nomogram plot was shown by“regplot” package. Calibration plots and decision curve analysis (DCA) were used to evaluate the efficacy (R package “caret” and “rmda”). Receiver Operating Characteristic (ROC) analyses were performed by “timeROC” R package. A dynamic nomogram was built from “rsconnect” and “DynNom” packages, enhancing the model's interactive visualization and utility for clinical decision-making.

### Tumor microenvironment analysis of programmed cell death score

The Tumor Immune Dysfunction and Exclusion (TIDE) algorithm was used to predict immunotherapy response across PCDS groups. Immune cells constitute a vital part of the tumor microenvironment (TME). Understanding the composition of immune cells within the tumor microenvironment (TME) is crucial for unraveling the intricate molecular mechanisms of cancer and guiding important clinical decisions 12. To quantify immune cell types in tumor samples, both marker-gene-based and deconvolution-based methods were implemented to enhance the reliability of the results, including Cell-type Identification By Estimating Relative Subsets of RNA Transcripts (CIBERSORT) 13, quanTIseq 14, Estimating the Proportion of Immune and Cancer cells (EPIC) 15, Microenvironment Cell Populations-counter (MCP-counter) 16, and TIMER 17. Stromal score, immune score, tumor purity, and ESTIMATE score were also computed through ESTIMATE algorithm. Relevant analyses were conducted with R package “immunedeconv”.

### Drug sensitivity prediction based on the prognostic signature

As for the prediction of common chemotherapy response, we estimated the half maximal inhibitory concentration (IC50) values of 5 best chemotherapy agents for UCEC based on the drug sensitivity data obtained from (GDSC) 18. R package “oncoPredict” was utilized to perform the above prediction of drug sensitivity.

### Statistical analyses

Statistical analyses were performed using R 4.2.0 (The R Foundation), unless otherwise stated. A comparison between two groups was made using either the Wilcoxon rank test or Student's t-test. The Kruskal-Wallis test was used for comparing multiple groups. Kaplan-Meier curves were generated to illustrate survival, and the log-rank test was employed for comparing the curves. All p-values were two-sided, and a significance level of p < 0.05 was considered statistically significant.

## Results

### Mutation landscape of programmed cell death genes in UCEC patients

**Figure 1:**
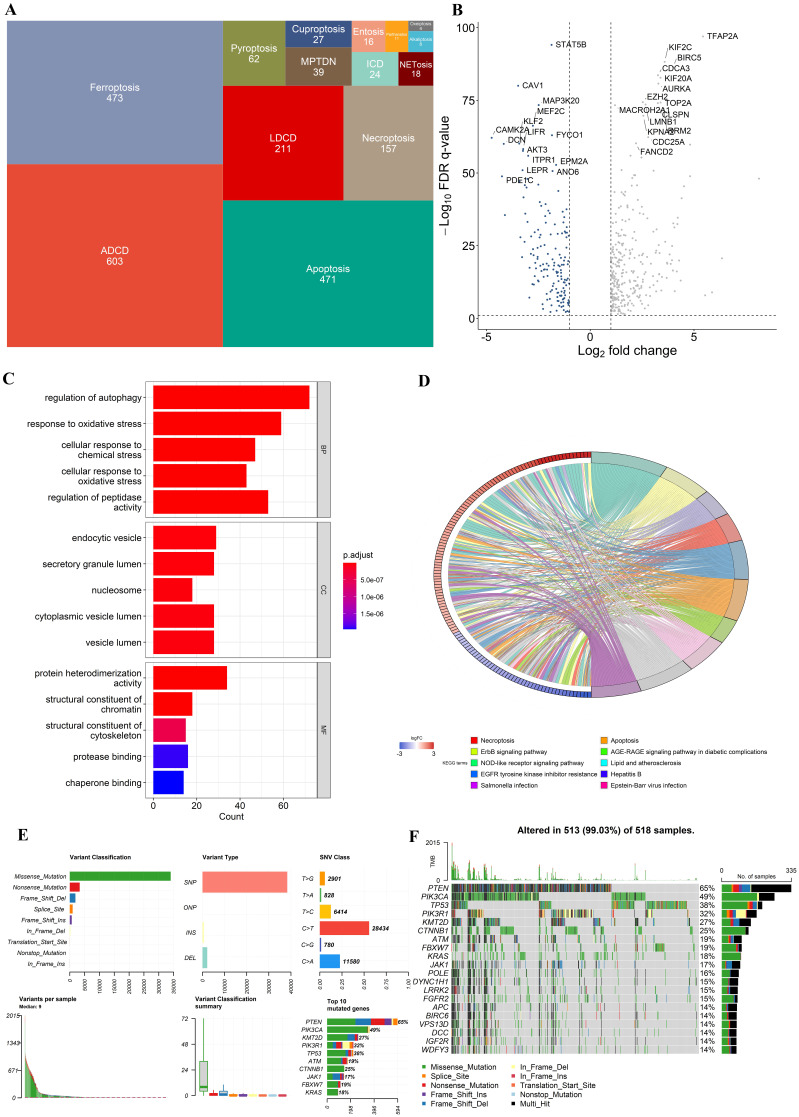
** Multi-omics landscape of programmed cell death genes in UCEC patients.** (A) The tree map of 14 related gene sets of programmed cell death. (B) Volcano plot of the PCD-related DEGs (green: down-regulated DEGs; red: upregulated DEGs; grey: not significant genes). (C) KEGG enrichment analyses based on the DEGs. (D) GO enrichment analyses based on the DEGs. (F) A summary plot and (G) an oncoplot of PCD-related genes in the TCGA-UCEC cohort.

In all, 603 ADCD genes, 473 ferroptosis genes, 471 apoptosis genes, 211 LDCD genes, 157 necroptosis genes, 62 pyroptosis genes, 39 MPTDN genes, 27 cuproptosis genes, 24 ICD genes, 16 entosis genes, 11 parthanatos genes, 8 alkaliptosis genes and 4 oxeiptosis genes were assembled (**Fig. 1A**). The genes of these different pathways intersected to some extent (**Supplementary Figure S1**). 1714 different genes were ultimately included in this study (**Supplementary Table S2**).

**Figure 2:**
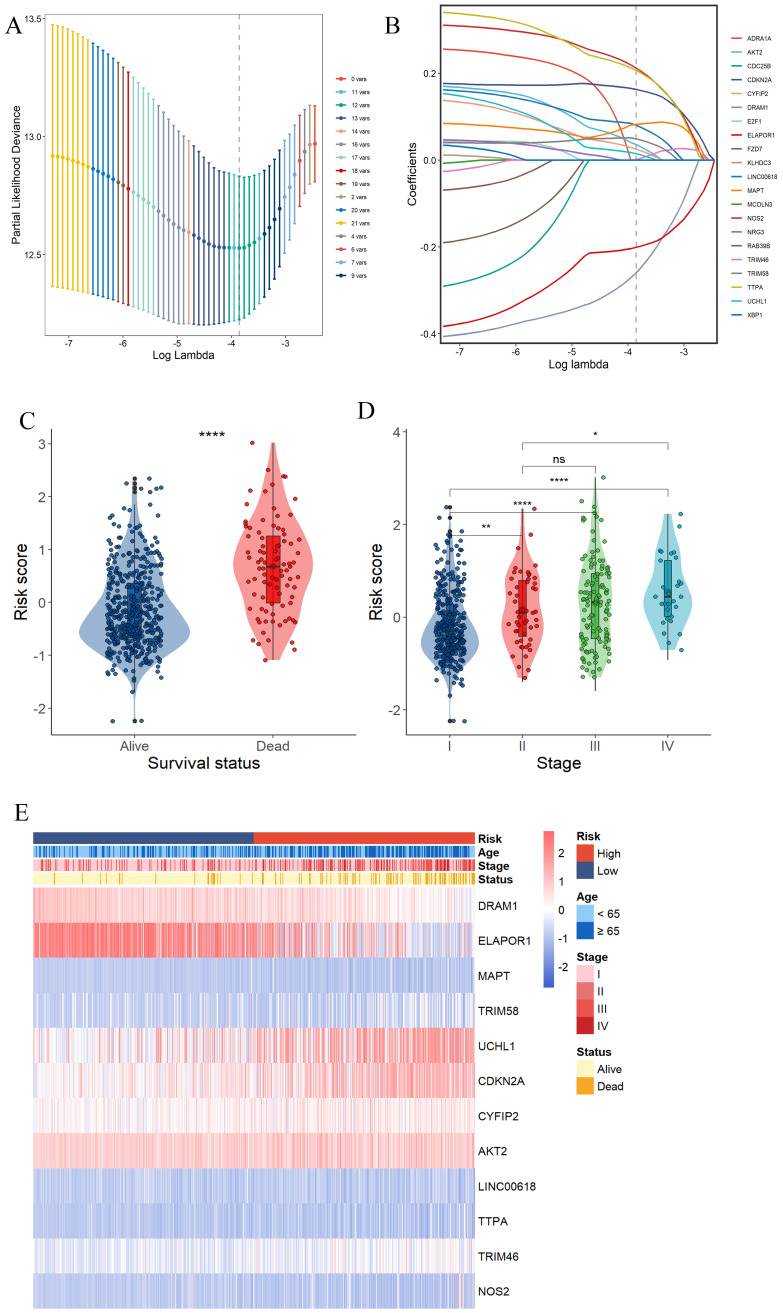
** Construction of a prognostic gene signature for UCEC patients.** (A) Selection of the 12 model genes by LASSO method. (B) Cross-validation of the constructed signature. (C) Violin plots of the relationship between PCDS and survival status. (D) Violin plots of the relationship between PCDS and clinical stage. **** means P < 0.0001; ** means P < 0.01; * means P < 0.05; ns means not significant. (E) Heatmap of 12 model genes and clinical features.

In the TCGA-UCEC cohort, we identified 460 differentially expressed genes (DEGs) (FDR q-value < 0.05, and |log2FC| > 1), with 302 upregulated and 152 downregulated between UCEC cases and controls. A volcano plot of the DEGs is presented in **Fig. 2B**. Besides, Kyoto Encyclopedia of Genes and Genomes (KEGG) and Gene Ontology (GO) enrichment analysis indicated that these DEGs were involved in multiple biological pathways such as regulation of autophagy, response to oxidative stress, necroptosis, etc. (**Fig. 2C and D**). The variation in PCD- related genes was also evaluated in UCEC patients from the TCGA cohort. Our result showed that approximately 99.03% (513/518) of UCEC patients had mutations, predominantly missense mutation (**Fig. 2F, G**). The top 20 mutations of PCD-related genes are displayed, with PTEN showing the highest mutation frequency (65%) and the others ranging from 14% to 49% (**Fig. 2F, G**).

### Construction of a prognostic gene signature for UCEC patients

Survival information of UCEC patients was collected for further analysis. The TCGA data were randomly divided into training and validation sets at a 2:1 ratio. Univariate Cox regression analysis was separately employed for the general screening of survival-related genes. A total of 19 genes (ADRA1A, DRAM1, ELAPOR1, MAPT, RAB39B, TRIM58, UCHL1, CDKN2A, NRG3, CYFIP2, CDC25B, AKT2, E2F1, LINC00618, TTPA, TRIM46, FZD7, NOS2, KLHDC3, XBP1, MCOLN3) in TCGA training dataset met the cutoff of FDR< 0.1.

**Figure 3:**
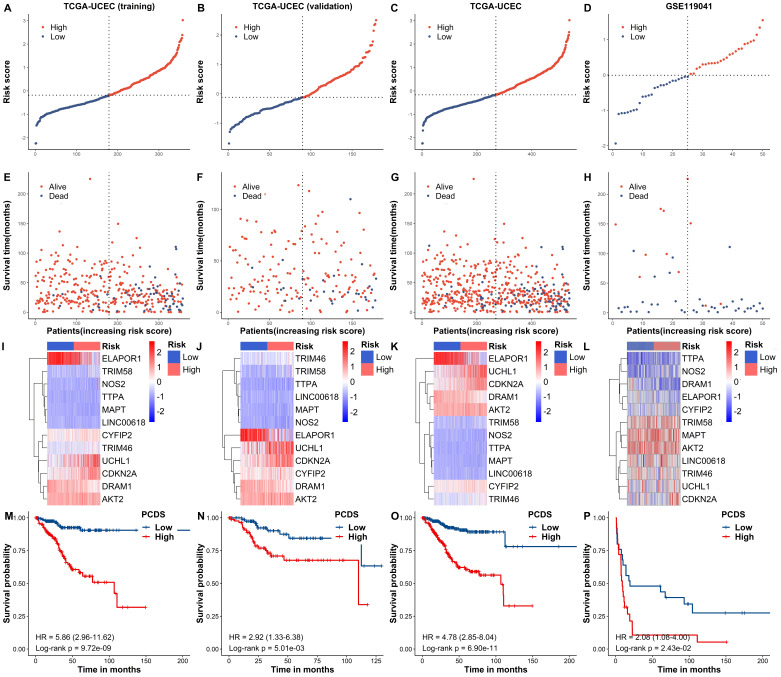
** Internal training and external validation of the gene signature in UCEC.** (A-H) Distribution of adjusted PCDS according to the survival status and time in TCGA (training), TCGA (validation), TCGA (combined), and GSE119041 cohorts. (I-L) Heatmap of PCD signature including 12 genes in TCGA (training), TCGA (validation), TCGA (combined), and GSE119041 cohorts. (M-P) KM curves of the low and high PCDS group patients in TCGA (training), TCGA (validation), TCGA (combined), and GSE119041 cohorts.

A 12-gene signature was constructed by LASSO-Cox regression analysis (**Fig. 3A and B**). Among them, CDKN2A was associated with Apoptosis, Cuproptosis and Ferroptosis. MAPT was associated with ADCD and apoptosis. 4 genes (DRAM1, ELAPOR1, TRIM58, UCHL1) were associated with ADCD. 2 genes (AKT2, CYFIP2) belong to Apoptosis. 4 genes (LINC00618, NOS2, TRIM46, TTPA) are associated with Ferroptosis. We investigated the correlation of each model gene and prognosis in the TCGA-UCEC using Kaplan-Meier analysis (**Supplementary Fig. 2**). All the model genes had a significant influence on the OS time (P < 0.05, **Supplementary Fig. 2**). Our model exported the programed cell death score (PCDS) of each patient by the formula: PCDS = -0.39*DRAM1-0.24*ELAPOR1+0.06*MAPT+0.07*TRIM58+0.05*UCHL1+0.2*CDKN2A+0.07*CYFIP2+0.09*AKT2+0.13*LINC00618+0.27*TTPA+-0.03*TRIM46+0.29*NOS2. PCDS was significantly associated with clinical features such as different UCEC survival status (alive or dead) and clinical stage (I-IV) (**Fig. 3C, D, E**).

**Figure 4:**
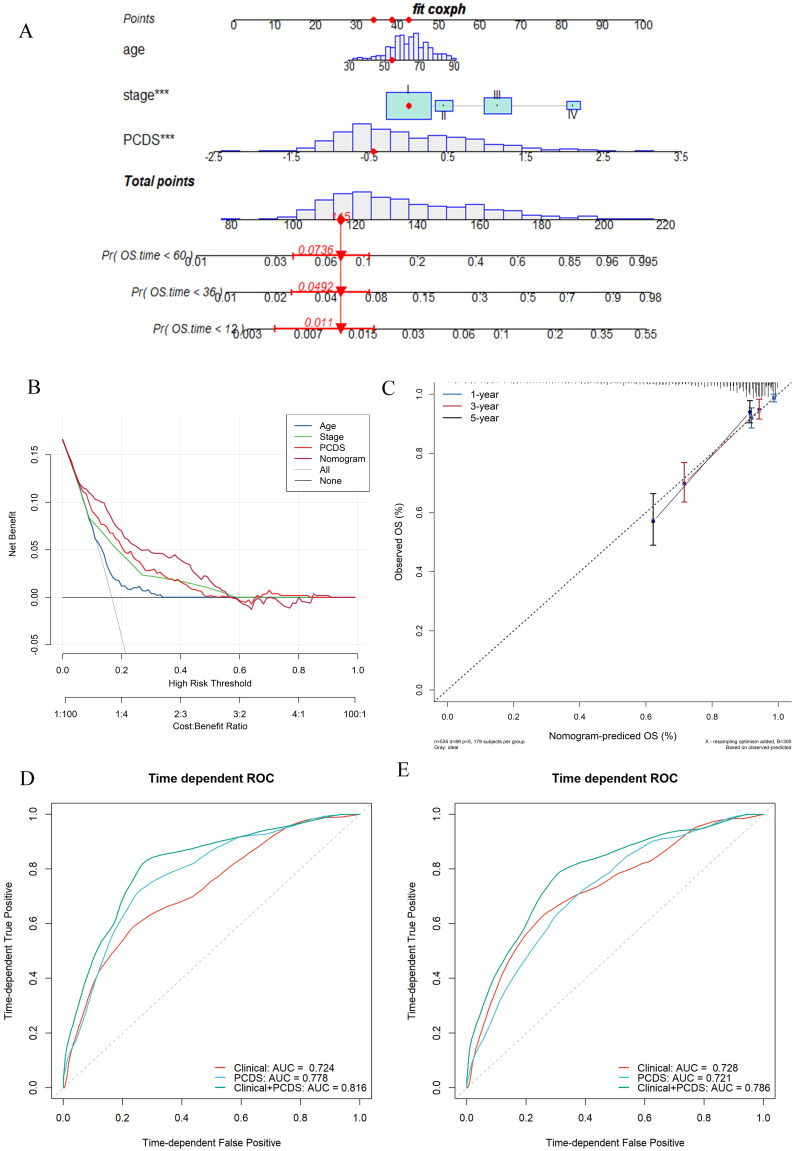
** Establishment and assessment of the nomogram survival model.** (A) A nomogram was established to predict the prognostic of UCEC patients. (B) Decision curve analysis (DCA) of nomogram predicting 5-year overall survival. (C) Calibration plots showing the probability of 3-, and 5-year overall survival in TCGA cohort. (D) 3- and (E)5-year overall survival of receiver operator characteristic (ROC) analysis of nomogram in TCGA.

Based on the median PCDSs, we differentiated UCEC patients in the TCGA cohort into a high-PCDS group and a low-PCDS group. Next, we compared the overall survival between groups. Our results revealed that patients with high PCDS had poor prognosis than those with low PCDS (**Fig. 4A-H**). The heatmap (PCA) showed that the classification was satisfying based on PCDS (**Fig. 4I-L**). A marked difference was detected in the OS time between these two groups; that is, patients in the low-PCDS group were more likely to have lower mortality rates (P < 0.05, **Fig. 4M-P**). Also, there exhibited a similar and good performance in the validation set of TCGA, the combined set of TCGA and an independent dataset of GSE119041.

### Establishment and assessment of the nomogram survival model

After adjusting for other confounding factors, the multivariate Cox analysis confirmed PCDS as an independent prognostic factor for UCEC patients (HR = 3.41, 95% CI: 2.06-5.66, P < 0.05, **Fig. 4A**). A nomogram model was developed using multivariable Cox and stepwise regression analyses in the TCGA cohort to estimate the 1-, 3-, and 5-year OS, incorporating age, stage, and PCDS (**Fig. 4A**). The C-index value of the model was 0.92 (95%CI: 0.86-0.97). Calibration curves showed the accuracy of this model in predicting the 1-, 3-, and 5-year survival rates (**Fig. 4C**). Moreover, we performed decision curve analysis (DCA) and found that the nomogram model was better than any other predictor applied in this study (**Fig. 4B**). Additionally, we evaluated the area under curve (AUC) values, and the results showed that the nomogram (clinical and PCDS) had high accuracy in predicting 3-, and 5-year survival of UCEC patients (AUC_3-year_=0.816, AUC_5-year_=0.786, **Fig. 4D, E**).

### Dissection of tumor microenvironment based on programmed cell

**Figure 5:**
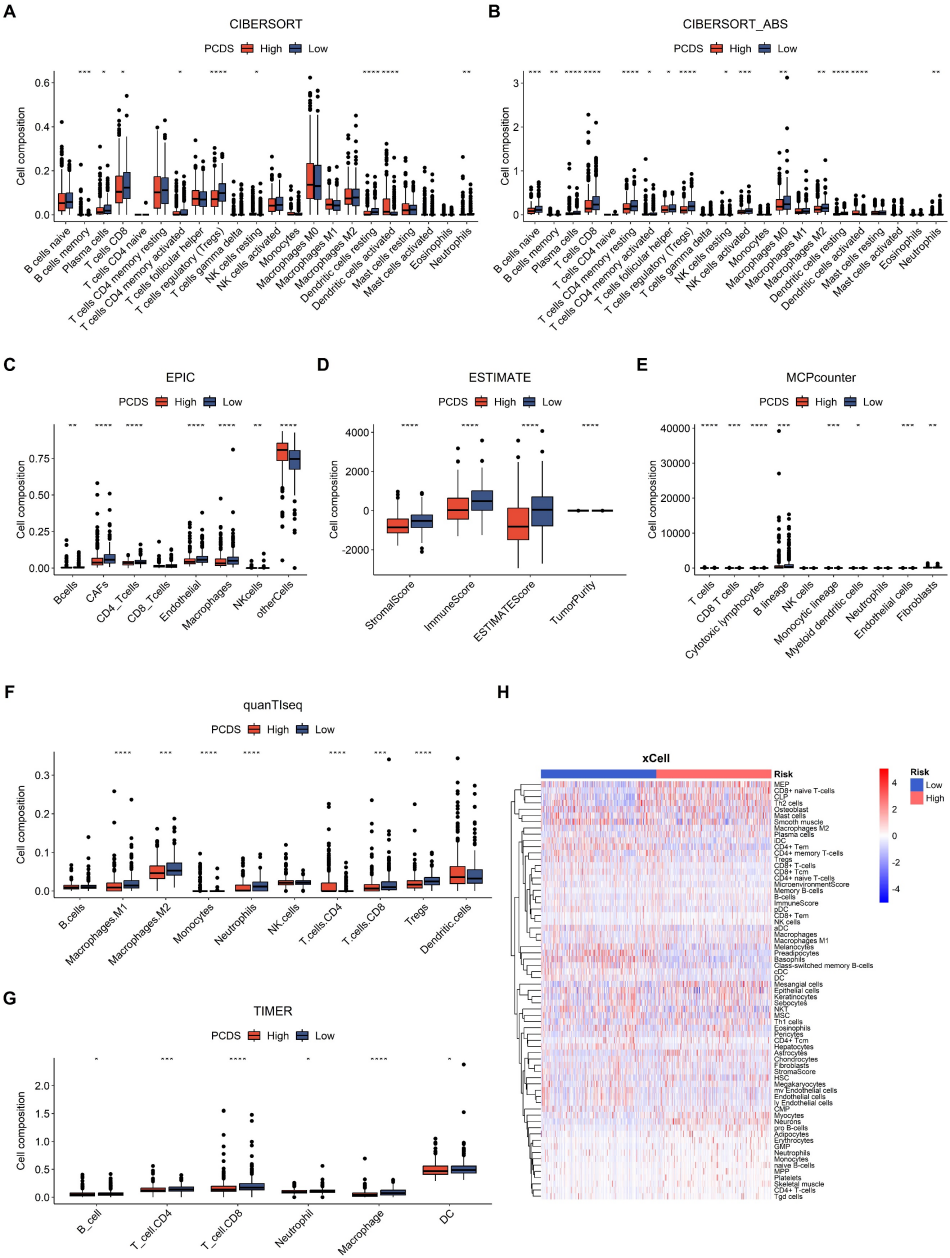
** Dissection of tumor microenvironment based on programmed cell death signature.** (A-G) Boxplots for different cell composition in low- and high-PCDS group patients UCEC patients. (H) Heatmap of all composition in low- and high-PCDS group patients by xCell method.

Furthermore, we used CIBERSORT, CIBERSORT-ABS, EPIC, ESTIMATE, MCP-COUNTER, QUANTISEQ, TIMER, and XCELL algorithms to measure the enrichment scores of immune-related cells and compare the differences between the two PCDS groups **(Fig. 5)**. Most of the immune cells showed differences in the high and low groups. In the ESTIMATE algorithm, we found that the high PCDS group had lower stromal, immune, and estimation scores.

### Efficacy of programmed cell death signature in predicting drug sensitivity

**Figure 6:**
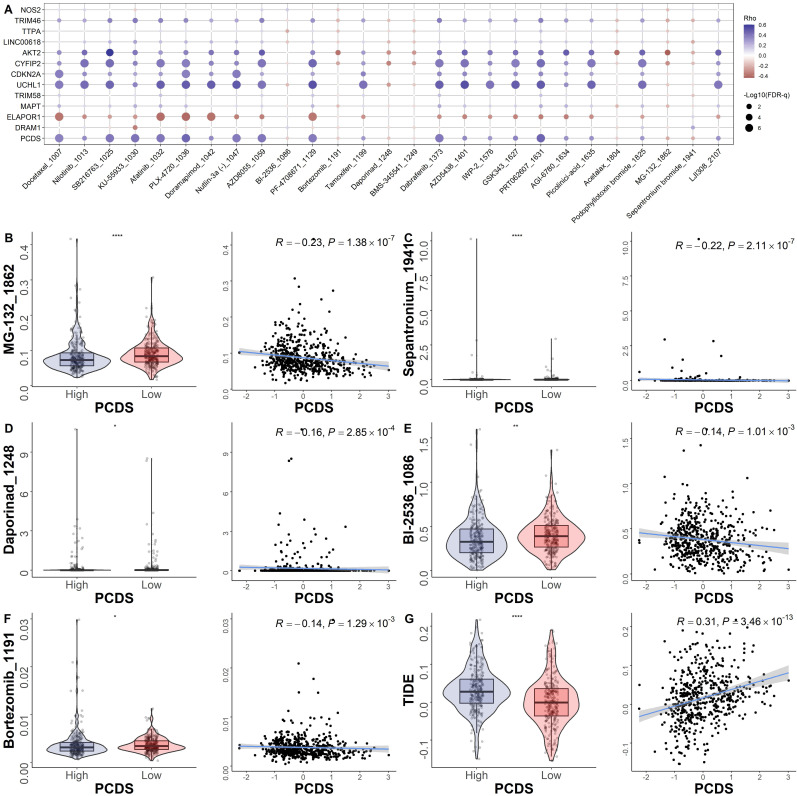
** Efficacy of programmed cell death signature in predicting drug sensitivity.** (A) Bubble plot of the relationship between drugs, PCDS, and model genes. (B-F) Boxplots of the comparison of IC50 of drugs between high- and low-PCDS groups, and correlation between the IC50 and PCDS in TCGA cohort. (G) Boxplots of the comparison of TIDE score between high- and low-CDI groups, and correlation between the TIDE score and PCDS values in UCEC patients. ** means P < 0.01; * means P < 0.05.

To explore the relationship between the model and drug sensitivity, we calculated each drug's half maximal inhibitory concentration (IC50) value in UCEC samples and identified significant differences between groups. The landscape of the correlation and significance between drug sensitivities and PCDS are showed in **Fig. 6A**. We found the IC50 values of MG-132, sepantronium, daporinad, BI-2536 and Bortezomib were lower in the high-PCDS group (**Fig. 6B-F**). These results suggest that UCEC patients with high-PCDS were sensitive to these drugs. Therefore, bortezomib has the potential to be used in the treatment of chemotherapy-resistant UCEC patients. The correlation between model genes and classical therapeutic targets in breast cancer is showed in **Fig. 6C**. Furthermore, we assessed the TIDE score in each UCEC patient. We found that the TIDE score was higher in the high-PCDS group and positively correlated with PCDS. This result indicates that UCEC patients with high PCDS may not benefit from immunotherapy (**Fig. 6D**).

## Discussion

The findings of this study provide valuable insights into the role of programmed cell death (PCD) pathways in the development and progression of Uterine Corpus Endometrial Carcinoma (UCEC). The dysregulation of PCD pathways has been implicated in various cancers, including UCEC. By analyzing thirteen PCD pathways, a novel 12-gene PCD signature was established and validated, demonstrating its effectiveness in predicting clinical prognosis in UCEC patients. The study revealed that UCEC patients with a high PCD score (PCDS) had a worse prognosis, indicating the potential clinical significance of PCD dysregulation.

Furthermore, the study explored the association between PCDS and the tumor microenvironment by diverse methods, suggesting potential implications for immunotherapy strategies in UCEC. Interestingly, patients with a high PCDS may not derive significant benefits from immunotherapy, highlighting the necessity for alternative treatments such as chemotherapy. Specifically, the study identified Bortezomib as a potential chemotherapy drug that may be effective in UCEC patients with high PCDS.

The establishment of this comprehensive PCD model enhances our understanding of the complex biological processes underlying UCEC. It provides a valuable tool for assessing prognosis and guiding treatment decisions in UCEC patients. The integration of PCD pathways into prognostic models holds promise for personalized medicine approaches, allowing for tailored treatment strategies based on individual patients' PCD profiles. Further research is warranted to validate these findings and explore the potential clinical applications of the PCD signature in UCEC management.

For these 12 PCD-related genes, many studies have shown that they play an important role in the development of cancer. Experimental studies showed that the NIVO-treated HT29 and HCT116 human colon cancer cells had down-regulated expression of DRAM1 19. ELAPOR1(also known as EIG121) is a good endometrial biomarker associated with a hyper estrogenic state and estrogen-related type I endometrial adenocarcinoma 20. Whole exome sequencing of DNA of 14 tumor tissue samples from endometrial cancer patients in Taiwan demonstrated MAPT as a potential driver gene and CYFIP2 as a potential passenger gene 21.

Through ubiquitination of DDX3, TRIM58 disrupts the p53/p21 pathway to enhance chemoresistance in breast cancer 22. Ubiquitin carboxyl terminal hydrolase L1 (UCHL1) belongs to the deubiquitinase (DUB) family of enzymes and is expired at high levels in various cancer types. Multiple significant studies have highlighted the emerging and crucial roles of UCHL1 in breast cancer 23. CDKN2A is associated with multiple forms of cell death and related to the origin of a variety of tumors 24-27. A retrospective study of patients with high-grade epithelial ovarian cancer showed that AKT2 amplification was associated with shorter PFS 28. A study showed a strong association between hepatitis B virus (HBV) and the integration of transmembrane homologous phosphatase, long intergenic non-protein coding RNA (LINC)00618, and ZBTB20 29. One study demonstrated that TRIM46 functions as a ubiquitin ligase targeting histone deacetylase HDAC1 for ubiquitination and subsequent degradation. This TRIM46-HDAC1 axis plays a crucial role in regulating a set of genes, particularly those involved in DNA replication and repair processes. As a result, TRIM46 promotes both breast cancer cell proliferation and chemoresistance in laboratory settings and significantly accelerates tumor growth *in vivo* 30. Experimental studies suggest that changes in NOS2 expression may help predict endometrial cancer progression and treatment outcomes 31.

The immune system plays a vital role in recognizing and eliminating cancer cells. It involves the activation of various immune cells, such as T cells, natural killer cells, and macrophages, which can recognize and destroy malignant cells. However, tumors often develop mechanisms to evade immune surveillance and suppress immune responses. Apoptosis plays a crucial role in maintaining tissue homeostasis by eliminating damaged or abnormal cells. In the context of UCEC, dysregulation of apoptotic pathways can contribute to tumor development and progression 32.

While our model demonstrated excellent performance in both the training and validation cohorts, it is important to acknowledge certain limitations. Firstly, the retrospective recruitment of patients may introduce some inherent bias to a certain extent. Secondly, more experiments are needed to be performed. Thus, additional validation through high-quality, multicenter randomized controlled trials with ample sample size and adequate follow-up is necessary.

In conclusion, a novel PCD model was established by comprehensively analyzing diverse cell death patterns. This model accurately predicts the clinical prognosis and drug sensitivity of UCEC. The findings suggest that the PCD signature can serve as a valuable tool in assessing prognosis and guiding treatment decisions for UCEC patients.

## Supplementary Material

Supplementary figures and tables.

## Figures and Tables

**Table 1:** Clinical information of TCGA-UCEC and GSE119041 cohort

Clinical parameters	TCGA-UCEC				GSE119041
	Overall	Training	validation	P value	N=50
	N=537	N=358	N=179		
OS (%)					
Alive	448 (83.4)	292 (81.6)	156 (87.2)	0.129	11(22.0)
Dead	89 (16.6)	66 (18.4)	23 (12.8)		39(78.0)
Survival time (median [IQR])	29.86 [16.85, 51.48]	28.91 [16.85, 51.38]	30.72 [16.90, 51.47]	0.531	11.71[5.02-60.85]
Age (mean (SD))	63.92 (11.17)	64.35 (10.84)	63.07 (11.80)	0.213	-
Race (%)					
White	368 (68.5)	247 (69.0)	121 (67.6)	0.866	-
Others	137 (25.5)	91 (25.4)	46 (25.7)		-
Unknown	32 (6.0)	20 (5.6)	12 (6.7)		-
menopause status (%)					
post	485 (90.3)	324 (90.5)	161 (89.9)	0.959	-
pre	52 (9.7)	34 (9.5)	18 (10.1)		-
stage (%)					
I	334 (62.2)	224 (62.6)	110 (61.5)	0.425	-
II	51 (9.5)	32 (8.9)	19 (10.6)		-
III	123 (22.9)	79 (22.1)	44 (24.6)		-
IV	29 (5.4)	23 (6.4)	6 (3.4)		-
