# Supplementary material for: Constructing a Prognostic Model of Uterine Corpus Endometrial Carcinoma and Predicting Drug-Sensitivity Responses Using Programmed Cell Death-Related Pathways
Source: J Cancer. 2024 Mar 31;15(10):2948–59. doi: 10.7150/jca.92201 (PMC11064274; doi:10.7150/jca.92201)
Supplement: Supplementary file 1 — Supplementary figures and tables. [file jcav15p2948s1.pdf]

Table S1. Gene collection of diverse programmed cell death pattern

Type	PCD	Database	MSigDB	GeneCards	KEGG	Reference
Autophagy-dependent cell death	ADCD	the Human Autophagy Database (HADb, http://autophagy.lu/clustering/index.html)	GOBP_REGULATION_OF_AUTOPHAGY<br>KEGG_REGULATION_OF_AUTOPHAGY<br>REACTOME_AUTOPHAGY<br>WP_AUTOPHAGY			
Alkaliptosis	Alkaliptosis					PMID:29248440
Apoptosis	Apoptosis		ALCALA_APOPTOSIS<br>GOBP_APOPTOTIC_SIGNALING_PATHWAY<br>HALLMARK_APOPTOSIS REACTOME_APOPTOSIS<br>WP_APOPTOSIS			
Cuproptosis	Cuproptosis	FerrDb V2 http://www.zhounan.org/ferrdb/current/				PMID:35627236
Entotic cell death	Entosis			GeneCards		
Ferroptosis	Ferroptosis	FerrDb V2 http://www.zhounan.org/ferrdb/current/				
Immunogenic cell death	ICD			GeneCards		
Lysosome-dependent cell death	LDCD			GeneCards		
MPT-driven necrosis	MPTDN					PMID:26956930
Necroptosis	Necroptosis		GOBP_NECROPTOTIC_SIGNALING_PATHWAY<br>GOBP_NECROPTOTIC_PROCESS<br>GOBP_POSITIVE_REGULATION_OF_NECROPTOTIC_PROCESS		hsa04217	
Netotic cell death	NETosis			GeneCards		
Oxeiptosis	Oxeiptosis			GeneCards		
Parthanatos	Parthanatos			GeneCards		
Pyroptosis	Pyroptosis		GOBP_POSITIVE_REGULATION_OF_PYROPTOSIS<br>GOBP_PYROPTOSIS<br>REACTOME_PYROPTOSIS			

Table S2. Differential PCD genes in TCGA-UCEC

Gene symbol	log2FC	p	q
AKT3	-3.23931	1.29E-60	2.46E-58
AQP5	4.002972	4.66E-35	2.08E-33
AURKA	3.288175	3.31E-84	1.86E-81
AVPR1A	-3.3601	3.94E-30	1.31E-28
BECN2	3.052972	4.35E-13	3.79E-12
BIRC5	3.997724	4.72E-90	3.61E-87
CA9	4.05934	1.23E-33	5.15E-32
CAMK2A	-4.74613	3.41E-65	7.79E-63
CAV1	-3.47002	1.83E-83	9.86E-81
CDCA3	3.266313	3.67E-87	2.30E-84
CDKN2A	4.816081	1.07E-51	1.26E-49
CLSPN	3.260604	2.16E-77	8.90E-75
CPEB1	-3.28098	4.29E-23	8.84E-22
CTSV	4.522571	3.87E-48	3.81E-46
DCN	-4.16487	4.73E-63	9.89E-61
DDR2	-3.06673	1.03E-47	9.92E-46
DPEP1	3.236434	1.60E-14	1.60E-13
DSG1	3.180434	8.01E-11	5.49E-10
DSG3	6.34832	1.10E-22	2.19E-21
DUSP1	-3.14798	1.55E-48	1.55E-46
E2F1	3.055965	1.26E-53	1.63E-51
EEF1A2	3.43074	3.79E-11	2.69E-10
EREG	3.687069	1.10E-15	1.22E-14
FGF21	4.07416	6.75E-11	4.67E-10
GDF15	3.201951	6.17E-33	2.46E-31
GPX2	4.17559	5.48E-18	7.48E-17
GSDMC	3.158456	1.17E-24	2.72E-23
H1-5	3.827943	4.78E-24	1.06E-22
H2AC12	3.714454	9.31E-21	1.59E-19
H2AC13	3.614795	1.56E-28	4.67E-27
H2AC14	3.987254	2.40E-18	3.38E-17
H2AC16	3.702553	4.16E-21	7.31E-20
H2AC17	3.942639	5.39E-22	1.01E-20
H2AC21	3.32772	4.42E-17	5.55E-16
H2AC4	3.881589	1.10E-20	1.88E-19
H2AC8	3.043733	4.05E-32	1.53E-30
KCNB1	-4.1097	5.66E-38	3.02E-36
KIF20A	3.388317	2.64E-86	1.61E-83
KIF2C	3.589597	6.49E-92	5.57E-89

KLF2	-3.41625	3.74E-63	7.89E-61
LCN2	3.027711	2.61E-16	3.09E-15
LEPR	-3.25968	8.37E-54	1.11E-51
LIFR	-3.22847	2.99E-61	5.88E-59
LINC01833	8.134324	7.72E-51	8.75E-49
MAGEA3	5.871599	1.85E-10	1.22E-09
MAGEA6	5.538443	2.12E-09	1.25E-08
MIOX	3.949624	4.88E-27	1.33E-25
MMP13	4.360432	1.03E-14	1.05E-13
MS4A15	3.984676	2.69E-18	3.77E-17
MT1G	4.204902	8.20E-35	3.62E-33
MT3	3.321089	6.60E-17	8.19E-16
MYCN	3.342105	1.48E-24	3.42E-23
NLRP7	3.459527	5.13E-16	5.91E-15
NQO1	3.182841	1.93E-25	4.75E-24
PDE1C	-4.24973	1.21E-51	1.41E-49
PDK4	-3.02072	1.46E-35	6.77E-34
PKP1	4.284671	6.81E-22	1.26E-20
PLA2G4F	4.221233	4.83E-37	2.42E-35
PLIN4	-3.16222	1.66E-28	4.95E-27
RRM2	3.539499	2.11E-75	7.66E-73
SFN	4.814932	8.63E-63	1.79E-60
SNCA	-3.12449	1.78E-38	9.73E-37
TERT	4.405616	6.99E-44	5.15E-42
TGFBR3	-3.2121	3.24E-44	2.44E-42
TIMP3	-3.40344	7.93E-50	8.42E-48
TOP2A	3.393847	1.30E-77	5.48E-75
TUBB3	3.303289	2.37E-27	6.58E-26
ZEB1	-3.02434	9.89E-51	1.11E-48

The genes with the absolute value of Log2FC >3 were kept.

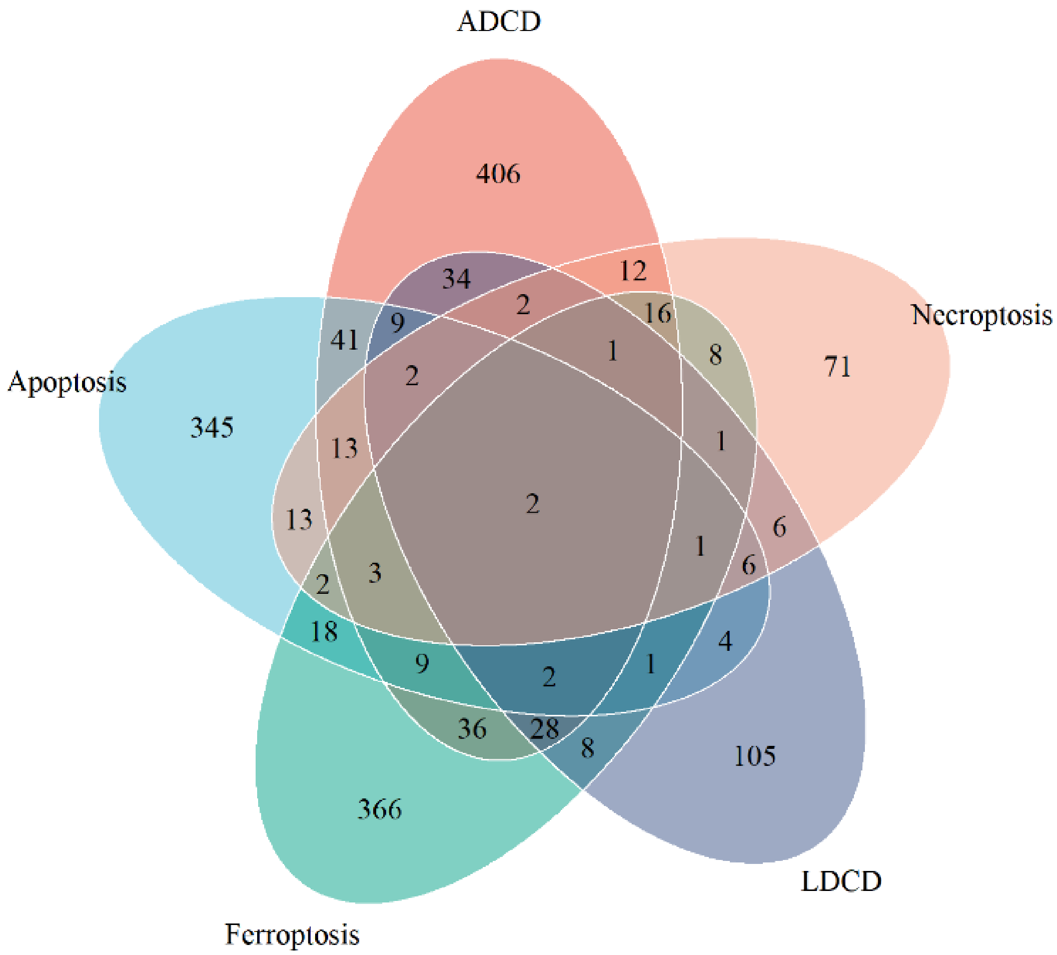

Figure S1. **Venn plot of PCD-pathway genes**

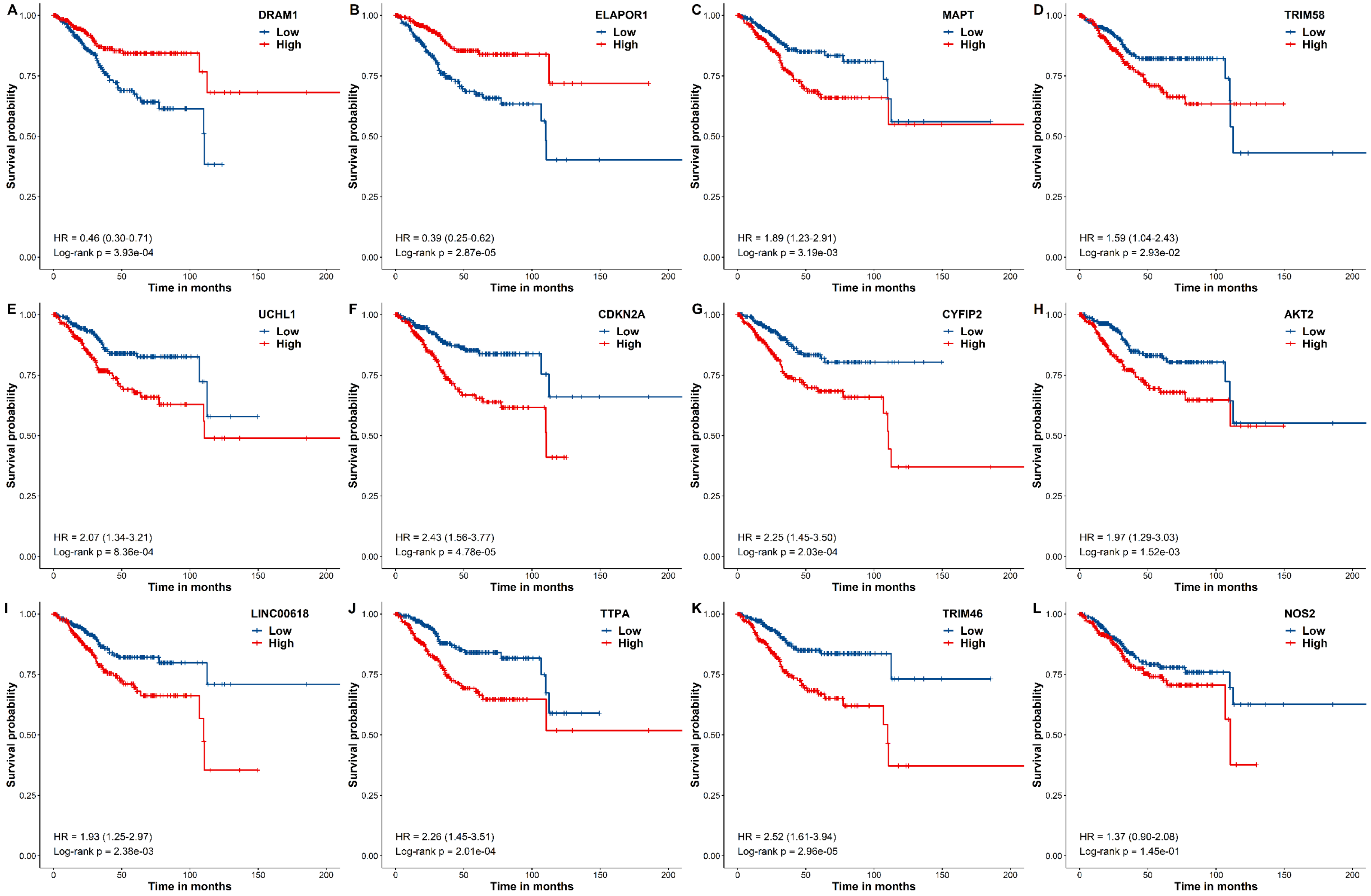

Figure S2. **KM curves of PCD signature genes**
